# Extracellular vesicle‐mediated crosstalk between NPCE cells and TM cells result in modulation of Wnt signalling pathway and ECM remodelling

**DOI:** 10.1111/jcmm.15129

**Published:** 2020-03-13

**Authors:** Natalie Lerner, Sofia Schreiber‐Avissar, Elie Beit‐Yannai

**Affiliations:** ^1^ Clinical Biochemistry and Pharmacology Department Ben‐Gurion University of the Negev Beer‐Sheva Israel

**Keywords:** exosomes, extracellular matrix, extracellular vesicles, non‐pigmented ciliary epithelium, primary open‐angle glaucoma, trabecular meshwork, Wnt

## Abstract

Primary open‐angle glaucoma is a leading cause of irreversible blindness, often associated with increased intraocular pressure. Extracellular vesicles (EVs) carry a specific composition of proteins, lipids and nucleotides have been considered as essential mediators of cell‐cell communication. Their potential impact for crosstalk between tissues responsible for aqueous humour production and out‐flow is largely unknown. The study objective was to investigate the effects of EVs derived from non‐pigmented ciliary epithelium (NPCE) primary cells on the expression of Wnt proteins in a human primary trabecular meshwork (TM) cells and define the mechanism underlying exosome‐mediated regulation that signalling pathway. Consistent with the results in TM cell line, EVs released by both primary NPCE cells and NPCE cell line showed diminished pGSK3β phosphorylation and decreased cytosolic levels of β‐catenin in primary TM cells. At the molecular level, we showed that NPCE exosome treatment downregulated the expression of positive GSKβ regulator‐AKT protein but increased the levels of GSKβ negative regulator‐PP2A protein in TM cells. NPCE exosome protein analysis revealed 584 miRNAs and 182 proteins involved in the regulation of TM cellular processes, including WNT/β‐catenin signalling pathway, cell adhesion and extracellular matrix deposition. We found that negative modulator of Wnt signalling miR‐29b was abundant in NPCE exosomal samples and treatment of TM cells with NPCE EVs significantly decreased COL3A1 expression. Suggesting that miR‐29b can be responsible for decreased levels of WNT/β‐catenin pathway. Overall, this study highlights a potential role of EVs derived from NPCE cells in modulating ECM proteins and TM canonical Wnt signalling.

## 1 INTRODUCTION

An estimated 8.4 million people globally are blind due to glaucoma, making it the second leading cause of irreversible blindness.^1^ This is projected to increase with rising life expectancy.^2^ The disease is characterized by progressive damage in the optic nerve fibres and characteristic visual field loss.^3^ Primary open‐angle glaucoma (POAG) is the predominant form of glaucoma in Western countries. Intraocular pressure (IOP) is the major modifiable risk factor for POAG, but other IOP‐independent risk factors may be involved in the pathogenesis of the disease.^4^

The control of IOP is dependent on the dynamic balance between aqueous humour (AH) production mainly by non‐pigmented ciliary epithelium (NPCE) and its out‐flow through the conventional and alternative pathways.^5^ In humans, IOP is primarily controlled by the out‐flow of the AH through the trabecular meshwork (TM), also known as the conventional out‐flow pathway. The TM is a porous structure composed of endothelial cells embedded in a dense extracellular matrix (ECM), composed of collagen, elastic fibres, proteoglycans and other macromolecules.^6^ Fibrotic changes or deposits of ECM into the TM can slow or block the out‐flow which leads to an elevation of the IOP.^7^, ^8^ Glaucoma is thus linked to TM structure and function where structural changes in the TM likely alter tissue architecture and biomechanical properties which, in turn, influence its resistance to AH out‐flow.^6^, ^8^

It has previously been proposed that crosstalk communication may exist between inflow tissue and out‐flow tissues through potential modulators in AH.^9^ Increasing numbers of papers describe the presence of many biologically active factors, signalling molecules, immune mediators and steroid hormones in the AH.^10^, ^11^, ^12^ Extracellular vesicles (EVs) were identified in AH and most biologically active compounds in the AH may be EV‐associated.^13^ Among EVs, exosomes are of particular interest because of their roles in cell‐to‐cell communication. Exosomes are 30‐150 nm nanoparticles that are released from most cell types under normal and pathological conditions.^14^ Nucleic acids and proteins are embedded in the phospholipid exosome membrane.^15^ These rich exosomal cargos have the potential to regulate gene expression, cell death, oxidative stress, cellular metabolism and signal transduction pathways.^16^ Once released, exosomes can be internalized by neighbouring or distal cells, and equip cells with the ability to influence cellular functions. The term extracellular vesicles will be used with agreement with the international society for extracellular vesicles recommendation.^17^, ^18^

Research on exosome and small EV function in the eye has been limited. Although the origin of AH EVs is presently unknown, we assume that their source may be from the NPCE cells, where AH is actively secreted. NPCE cells contain a wide range of secretory vesicles in their cytoplasm, ranging from 10 nm to up to 1.5 μm in diameter.^9^ Smaller‐sized vesicles (10‐100 nm) include translucent and dense‐core secretory vesicles and may possibly represent EV‐like nanoparticles. Previously, we provided evidence that NPCE cells secrete EVs that were internalized specifically by TM cells in culture. In addition, we demonstrated that incubation of TM cells with NPCE‐derived EVs induced a significant decrease in the expression of the Wnt signalling pathways proteins and genes in human NPCE and TM cell lines.^19^

The Wnt signalling pathway, one of the best‐studied signalling cascades, has been implicated in POAG disease. While the underlying mechanism is still unclear, addition of exogenous sFRP1, a specific Wnt/β‐catenin signalling inhibitor, to ex vivo perfusion cultured eyes, decreased out‐flow facility and increased the IOP.^20^ This active Wnt signalling is found to be involved in TM cell–mediated ECM expression and TM cell stiffening.^21^ Overall, Wnt signalling appears to be a key player in TM regulation, with strong evidence suggesting that abnormal Wnt signalling may promote IOP.

Wnt signalling is known to crosstalk with other signalling mechanisms including Notch, NF‐κB, mTOR, transforming growth factor β, protein kinase C and PI3K/AKT.^22^ Among them, PI3K/AKT has been shown in several investigations to act upstream of Wnt signalling, resulting in β‐catenin stabilization via phosphorylation on Ser 9 of GSK‐3β .^23^ Previous studies have supported a molecular intersection between components of the Wnt and PI3K/Akt signalling pathways.^24^ Induced IOP elevation in rats has been shown to activate the PI3K/Akt pathway.^25^

Along the cascade of the PI3K/AKT signalling pathway, an additional potential element involved in the regulation of the Wnt pathway is protein phosphatase 2A (PP2A). PP2A has been shown to negatively regulate Wnt pathway.^26^, ^27^ PP2A was found to be significantly higher in AH samples from the POAG group as compared to a cataract group.^28^ After treatment with DEX, TGFβ2 alkaline phosphatases activity was reported to be increased in TM cells. Alkaline phosphatase activity was also found to be significantly higher in intact TM tissues from glaucomatous patients.^29^

In the present study, we turned to a primary cell model and investigated the effects of EVs obtained from human NPCE primary cells on primary human TM cells, with emphasis on the Wnt signalling pathway. Based on the findings that NPCE and TM cell lines establish a EV‐mediated crosstalk leading to the inhibition of Wnt signalling in TM cells, we expected that primary NPCE EVs would also suppress the Wnt signalling pathway in primary TM cells. In addition, to gain further mechanistic insight into this phenomenon, we analysed whether EVs derived from NPCE cell line might alter expression of proteins involved in the regulation of Wnt signalling cascades including AKT and PP2A proteins. Finally, to better understand the role of NPCE EVs in NPCE‐TM communication, we also evaluated miRNA and proteome profile of the NPCE‐derived EVs.

## 2 MATERIALS AND METHODS

### 2.1 Cell culture

A human normal trabecular meshwork (TM) cell line was generously donated by Alcon Laboratories, Texas, USA. A human non‐pigmented ciliary epithelial cell line was kindly supplied by Prof. Miguel Coca Prados, Yale University, MA, USA. A human retinal pigment epithelium (RPE) cell line was a gift from Dr Zeev Dvashi, Kaplan Medical Center, Rehovot, Israel. All cell lines were cultured in high glucose Dulbecco's modified Eagle's medium (DMEM) with 10% FBS (foetal bovine serum), 2 mmol/L L‐glutamine, 100 μg/mL streptomycin and 100 units/mL penicillin (all from Biological Industries, Kibbutz Beit HaEmek, Israel) in a humidified atmosphere of 95% air and 5% CO_2_ at 37°C. Primary TM cells were kindly provided by W. Daniel Stamer, Duke Eye Center, Durham, North Carolina, USA. Primary TM cells used in these experiments originated from a 63‐year‐ old individual marked as HTM138.2 (passage 2). Primary TM cells were cultured in low glucose DMEM containing 10% FBS, glutamine and antibiotics. Primary human NPCE was purchased from ScienCell Research Laboratories. Primary NPCE cells were maintained in epithelial cell medium (EpiCM; ScienCell Research Laboratories) supplemented with 2% FBS, 1% epithelial cell growth supplement (EPiCGS; ScienCell Research Laboratories) and 1% penicillin‐streptomycin solution (P/S; ScienCell Research Laboratories).

### 2.2 EV isolation

Dulbecco's modified Eagle's medium with EV‐depleted FBS was prepared by ultracentrifugation of culture medium for 16 hours at 110 000 *g* overnight at 4°C using a SW28 rotor followed by filtration through a 0.22 µm filter (*Millipore* Express PLUS (*PES*) membrane). EVs were isolated from conditioned media (CM) of cultured NPCE cell line or from CM of Primary NPCE cells using a polyethylene glycol (PEG) based approach as previously described.^30^, ^31^ Supernatant was collected from cells that were grown in medium containing EV‐depleted FBS for 48 hours and was subjected to centrifugation steps at 300 *g* for 10 minutes and 2000 *g* for 10 minutes to remove cell debris and larger vesicles. The resulting supernatant was filtered using 0.2 µm filter and added to the filtered precipitation solution (50% PEG8000 (Sigma), 0.5 mol/L NaCl in PBS), to achieve final PEG concentration (8.3% w/v), and samples were then mixed by repeated inversion and incubated overnight at 4°C. EVs were precipitated by centrifugation at 1500 *g* for 30 minutes, and the supernatant was removed. Residual fluid centrifugation was eliminated by centrifugation at 1500 *g* for 5 minutes and pelleted EVs were resuspended in PBS for further analysis.

### 2.3 Transmission electron microscopy at cryogenic temperature (cryo‐TEM)

For cryo‐TEM, 4 µL of the vesicle suspension was applied to a copper grid coated with a perforated lacy carbon 300 mesh (Ted Pella Inc) and blotted with filter paper to form a thin liquid film of solution. The blotted sample was immediately plunged into liquid ethane at its freezing point (−183°C) in an automatic plunger (Lieca EM GP). The vitrified specimens were transferred into liquid nitrogen for storage. Sample analysis was carried out under a FEI Tecnai 12 G2 TEM, at 120 kV with a Gatan cryo‐holder maintained at −180°C. Images were recorded with the Digital Micrograph software package, at low‐dose conditions, to minimize electron beam radiation damage. The measurements were performed at the Ilse Katz Institute for Nanoscale Science and Technology Ben‐Gurion University of the Negev, Beer Sheva, Israel.

### 2.4 EV size and concentration by Tunable Resistive Pulse Sensing (TRPS)

Extracellular vesicle concentration was determined by qNano (Izon Science) instrument, using the Tunable Resistive Pulse Sensing (TRPS) principle. This principle enables reception of signal while a single particle transfers through the NP150A membrane with pores of 150 nm. In order to eliminate contaminating debris, EV samples were passed through 0.22 μm filters. The apparatus was operated at a voltage of 0.48 V‐0.64 V and a pressure equivalent to 8 cm of H_2_O. The membrane was stretched to 45‐47 mm. Polystyrene beads at a concentration of 1 × 10^13^ beads/mL (114 nm; CPC100 Izon Science) were used to calibrate size and concentration, following the manufacturer's instructions. Samples were diluted 1000‐fold with PBS buffer and measured over 10 minutes. The movement of the particle through the membrane was identified as change in the ionic stream causing voltage changes. The power of the signal is proportional to the particle size. According to the amount of particles and their velocity at a specific time, the qNano determines the EVs’ concentration.

### 2.5 Western blotting

Western blotting analysis was performed on primary TM cells to ascertain the effects of NPCE EVs on Wnt signalling. Primary TM cells (1 × 10^6^ cells/well) were seeded in 6‐well plates in low glucose DMEM, containing 10% EV‐depleted FBS. After 24 hours, primary NPCE‐derived EVs (1 × 10^9^ particles) were added to the primary TM cells for 1, 2, 4, 6 and 24 hours. Untreated cells were used as negative control and EVs isolated from NPCE cell lines were used as positive control. After the indicated incubation time, primary TM cells were lysed with lysis buffer (20 mmol/L HEPES, 150 mmol/L NaCl, 1 mmol/L EGTA, 1 mmol/L EDTA, 10% glycerol, 1 mmol/L MgCl_2_, 1% Triton X‐100) containing a protease inhibitor cocktail. Whole cell lysates were separated on 10% polyacrylamide‐SDS gels and transferred to nitrocellulose membranes. After blocking (5% BSA, 1 hour at room temperature), membranes were incubated overnight at 4°C with phospho‐GSK3β (1:1000, Cell Signaling, 9323) and β‐catenin (1:1000, Cell Signaling, 8480). Then, membranes were incubated with secondary antibody solutions for 1 hour at room temperature with IgG‐horseradish peroxidase (HRP)–conjugated donkey anti‐rabbit antibody (1:5000; GE Healthcare). Antigen‐antibody complexes were detected using ECL Western blotting detection reagents (Biological Industries), followed by exposure to FUJI X‐ray film (Fuji medical X‐ray film, FujiFilm). Protein load was normalized by β‐actin protein level measurements using mouse anti‐β‐actin antibody (1:4000, Sigma‐Aldrich) followed by exposure to horseradish peroxidase‐conjugated goat antimouse antibody (1:20 000, Jackson ImmunoResearch Inc). After completion of the control experiments with primary cells to strengthen the findings seen in cell lines, we further examined whether NPCE cell line‐derived EVs modulate the expression of key proteins involved in Wnt regulation using the TM cell line. Western blot analysis was performed on protein lysates from TM cell line (1 × 10^6^cells/well in 6‐well plates) either untreated (Ctrl) or treated at various time‐points (1, 2, 4, 6, 8 and 24 hours) with NPCE cell line‐derived EVs (1 × 10^9^ particles) or RPE derived EV control (1 × 10^9^ particles). The nitrocellulose membranes were then incubated overnight at 4°C with a primary antibody against one of the following proteins: Anti‐PP2A (1:10 000 BD Biosciences*, *610555;) phospho‐Akt Ser473 (1:1000, Cell Signaling, 4060), phospho‐Akt Ser308 (1:1000, Cell Signaling, 13038), phospho‐PDK1 (1:1000, Cell Signaling, C49H2) and phospho‐mTOR (1:10 000, *Santa Cruz* Biotechnologies, sc‐293133). Total proteins were determined using antibodies to total Akt (1:1000, *Cell Signaling*, *4691*) and mTOR (1:10 000, *Santa Cruz* Biotechnologies, sc‐293089). To evaluate the effect of the NPCE EVs on collagen expression, the TM cell line was treated with NPCE cell line EVs for 24 hours, and 20 µg of protein extracted from TM cells was separated on 7.5% polyacrylamide‐SDS gels and probed for COL3A1 Antibody (1:1000, *Santa Cruz* Biotechnologies, sc‐271249).

### 2.6 Sucrose density gradients

Exosome pellets were resuspended in 2ml HEPES/sucrose (2.5 mol/L sucrose in 20 mmol/L HEPES) and loaded onto a continuous sucrose gradient (0.25‐2.0 mol/L in 20 mmol/L HEPES, pH 7.2) prepared using a Hoefer SG30 gradient maker. The gradients were centrifuged overnight at 210 000 *g* at 4°C in a SW‐41 swinging bucket rotor. 1 mL fractions were collected from the top to bottom of each gradient and 50 μL aliquots were used for density determination. The refractive index of each fraction was measured at 22°C using an Abbe refractometer (Atago 1211 NAR‐1T liquid) and converted to density using the conversion table published in the Beckman Coulter ultracentrifugation manual. Next, each fraction was washed in 20 mmol/L HEPES, ultra‐centrifuged at 100 000 *g* for 1 hour and mixed with lysis buffer prior to analysis by Western blotting.

### 2.7 Image stream analysis

Cellular uptake of the NPCE RNA and proteins via EVs was analysed using image stream. TM cells (5 × 10^5^) were seeded in a 6‐well plate in high glucose DMEM, containing 10% EV‐depleted FBS. After 24 hours, the TM cells were incubated for 2 hours with Exo‐Red (EXOC300A‐1, SBI, CA, USA) or Exo‐Green (EXOC300A‐1, SBI) stained EVs isolated from NPCE cells. Labelling of exosomal RNA by Exo‐Red and proteins by Exo‐Green was performed according to the manufacturer's protocol. 10 μL of Exo‐Red or Exo‐Green was added to 20 μg of EVs resuspended in 500 μL 1×PBS. The EV suspension was mixed with the stain solution and incubated for 10 minutes at 37°C. The labelling reaction was stopped by adding ExoQuick‐TC reagent. Labelled EVs were placed on ice for 30 minutes following centrifugation at 17136 *g* for 3 minutes. 100 μL of labelled NPCE EVs were co‐cultured with TM cells for two hours washed and analysed on the ImageStreamX Mark II imaging cytometer equipped with 60× objective. Exo‐Green and Exo‐Red fluorescence were recorded using excitation with 488 nm and 642 nm lasers, respectively at 3 mW intensity.

### 2.8 Microarrays analysis

Isolation and purification of RNA from NPCE cell line EVs were carried out using a miRCURY RNA isolation kit (Exiqon, Woburn, MA) according to the recommended procedure. RNA concentration and purity were determined at 260 nm by a NanoDrop2000 spectrophotometer (Thermo Scientific). RNA quality was assessed using an Agilent 2100 Bioanalyzer (Agilent Technologies). miRNA expression analyses* *were* *performed* *at the Life Sciences Core Facilities, Israel Weizmann Institute* *of Science using SurePrint G3 Human miRNA 8 × 60k microarrays (Agilent Technologies) according to the manufacture's protocol. Hybridization signals were detected with a DNA Microarray Scanner (Agilent Technologies), and the intensity of each hybridization signal was evaluated using Agilent Feature Extraction software.

### 2.9 Sample preparation and LC‐MS

For mass spectrometry, initially EVs were isolated by ultracentrifugation and the resulting pellet was resuspended in HPLC‐grade water. 100 µg of the exosome sample was dried in a speedvac and dissolved in 7.2 mol/L urea and 100 mmol/L ammonium bicarbonate. Disulphide bonds were reduced by adding 5 µL of 45 mmol/L 1,4‐dithiothreitol (DTT) for 15 minutes at 56ºC and alkylated with 100 mmol/L iodoacetamide for 15 minutes at room temperature in the dark. Subsequently, sequencing‐grade trypsin (Promega) was added to a final 1:50 w/w trypsin‐to‐protein concentration and digestion was performed overnight at 30°C. Proteolysis was stopped by adding 10 µL of ultra‐pure glacial acetic acid. Resulting peptides were dried by speedvac to evaporation the solvent and re‐dissolved in a 10 µL mixture of 5% acetonitrile and 0.1% formic acid. LC/MS analysis of digested peptides was performed using an Eksigent nano‐HPLC (model nanoLC‐2D) connected to the LTQ Orbitrap XL ETD (Thermo Fisher Scientific). Reverse‐phase chromatography of peptides was performed using a homemade C‐18 column (15 cm long, 75 μm ID) packed with Jupiter C18, 300 Å, 5 μm beads (Phenomenex). Peptides were separated by a 70‐minutes linear gradient, starting with 100% solvent A (5% acetonitrile, 0.1% formic acid) and ending with 80% solvent B (80% acetonitrile, 0.1% formic acid), at a flow rate of 300 mL/min. A full scan, acquired at 60 000 resolutions, was followed by CID MS/MS analysis performed for the five most abundant peaks, in the data‐dependent mode. Fragmentation and detection of fragments were carried out in the linear ion trap. Maximum ion fill time settings were 500 ms for the high‐resolution full scan in the Orbitrap analyzer and 200 ms for MS/MS analysis in the ion trap. The AGC settings were 5x10^5^ and 1 × 10^4^ (MS/MS) for Orbitrap and linear ion trap analyzers, respectively. Proteins were identified and validated using the SEQUEST and Mascot search engines operated under the Proteome Discoverer 1.4 software (Thermo Fisher Scientific). Mass tolerance for precursors and fragmentations was set to 10 ppm and 0.8 Da, respectively. Only proteins containing at least two peptides of high confidence (Xcore N2 or 2.5 for doubly or triply charged species, respectively) were chosen.

### 2.10 Statistical analysis

Data are presented as the means ± SD. Data were analysed using two‐way ANOVA followed by Bonferroni's post‐test. Statistical analysis was performed using GraphPad Prism version 5 software (GraphPad Software, Inc). Differences were considered statistically significant at *P* < .05.

## 3 RESULTS

### 3.1 NPCE EV traffic RNA and proteins between NPCE and TM cells

**Figure 1:**
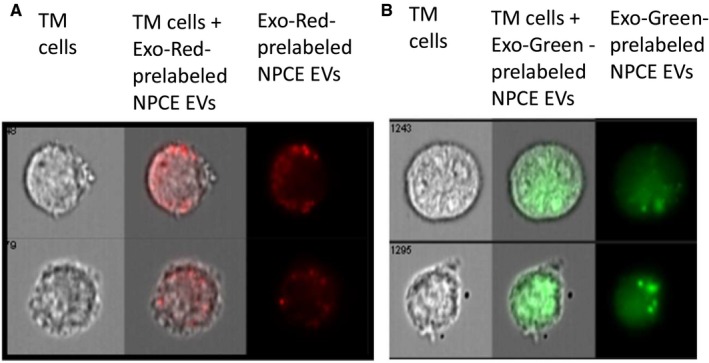
TM cells uptake of NPCE EVs. NPCE EVs in which either RNAs were labelled with Exo‐Red or proteins were labelled with Exo‐Green dyes co‐incubated for 2 h with TM cell line. EV fluorescence was captured and photographed using an ImageStreamTM high‐resolution imaging flow cytometer. A, Representative ImageStreamTM images showing Exo‐Red labelled NPCE EVs or B, Exo‐Green labelled NPCE EV uptake by TM cells

To study whether encapsulated RNAs and proteins in NPCE EVs are transferred to TM cells, we stained exosomes with Exo‐Red (RNA dye) or Exo‐Green (protein dye). These were then added to the TM cells for 2 hours. Cell images were then collected by Image Stream Flow Cytometry. After 2 hours, both the Exo‐Red (Figure 1A) and Exo‐Green (Figure 1B) signals were detected in the cytoplasm of TM cells exposed to these labelled EVs. These results suggest that NPCE EVs were internalized by TM cells and effectively delivered their cargo into the TM cytosol.

### 3.2 Characterization of exosomal proteins by LC‐MS/MS

We further performed protein analysis of NPCE EVs derived from culture media to identify proteins that can potentially function as mediators of cell‐to‐cell signalling. Using the LC‐MS technique, the combination of three independent exosome preparations resulted in the identification of 182 proteins. The 91 proteins detected in all three samples are shown in Table 1. This list included proteins known to be involved in cell adhesion, cytoskeleton regulation, oxidative stress response and others. Several of the identified proteins are associated with membrane trafficking and fusion processes. An assortment of cytosolic enzymes and various membrane transporters were also present. The list of proteins identified in this study is broadly consistent with that of other exosome proteome studies, further supporting the enrichment of EVs in samples prepared from NPCE supernatants. Along with our hypothesis that NPCE EVs participate in signalling within the drainage system, we also identified cellular signal transduction related proteins that were described previously as regulators of the Wnt signalling pathway (14‐3‐3 protein and histidine triad nucleotide‐binding protein 1).^32^, ^33^ In addition, our proteomic analysis revealed the presence of CD9 in EVs released by NPCE cells. CD9, a member of the tetraspanin family, has been implicated in inhibition of Wnt/β‐catenin signalling through the reduction of the cellular pool of β‐catenin.^34^ These results suggest that EVs carry proteins involved in Wnt signalling regulation, and the presence of these proteins further supports the idea that EVs may facilitating crosstalk between NPCE and TM cells by transferring their contents to target cells.

**Table 1:** List of proteins identified by LC‐MS/MS analysis in NPCE exosomes

Adhesion	CD44, CD59, CD9, integrin α, β, EDIL3
Antigen presentation	β‐2‐microglobulin, HLA class I histocompatibility antigen
Chaperons	Hsc70, Hsp71, Hsp90, T‐complex protein‐1 ε
Cytoskeleton	Actin α, β, α‐actinin, cofilin, collagen, ezrin, filamin, keratin, moesin, myosin‐6,9, prelamin, profilin, talin, tropomyosin α, tubulin α, β, vimentin, vinculin
Enzymes	ATPase NA+/K+ transporting, α‐enolase, D‐3‐phosphoglycerate dehydrogenase, G6PD, glyceraldehyde‐3‐phosphate dehydrogenase, fructose‐bisphosphate aldolase, L‐lactate dehydrogenase, malate dehydrogenase, nucleoside diphosphate kinase, phosphoglycerate mutase, phosphoglycerate kinase, pyruvate kinase, peptidylprolyl isomerase, protein disulphide‐isomerase, transitional endoplasmic reticulum ATPase, triosephosphate isomerase, ubiquitin carboxyl‐terminal hydrolase isozyme, valosin‐containing protein
Lectin binding	Galectin‐1
Vesicles trafficking & fusion	Annexins (1,2,5,6), clathrin heavy chain, Rab GDI, Rap 1B, WD RCP1
Others	Basigin, BASP1, calmodulin, importin, histones (H1.2, H1.5, H2A, H2B, H4), transgelin, protein DJ‐1, S100 protein
Oxidative stress response	Glutathione S‐transferase, peroxiredoxin‐1, superoxide dismutase
Protein synthesis	Initiation factor 4A, elongation factor (1,2), ribosomal proteins (40 S, 60 S subunit)
RNA/ DNA binding	Heterogeneous nuclear ribonucleoprotein, histidine triad nucleotide‐binding protein 1, nucleophosmin
Signal transduction	14‐3‐3 (ε, η, ζ), Rac (1,2,3), G proteins
Transporters	SLC3A2, chloride intracellular channel protein 4

### 3.3 NPCE exosome miRNA characterization

**Figure 2:**
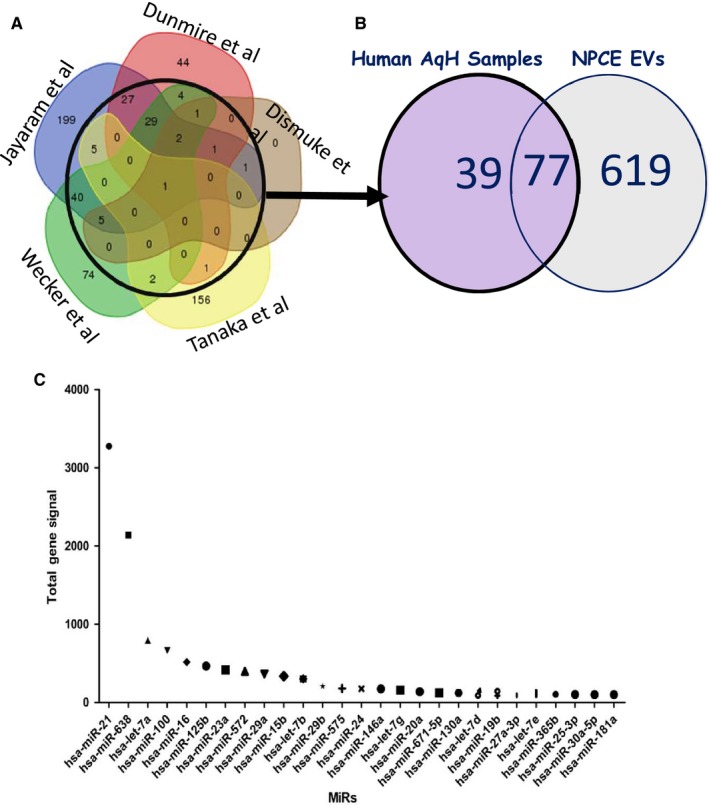
A comparison of miRNAs detected in the NPCE samples to the most overlapping miRNAs families in the AH. A, The Venn diagram adapted from Hari Jayaram et al^53^ depicts the overlap of human AH miRNAs detected in five studies. B, Venn diagram displays the total number of miRNAs detected in the EV samples isolated from NPCE cell culture medium (n = 2) compared to the most overlapping AH miRNAs families from the published datay.^53^ C, Expression levels of 27 miRNAs identified in EVs derived by NPCE cell line overlapping with those observed within the AH

We demonstrated from the proteomics experiments the presence of RNA related proteins in EVs isolated from NPCE cells. Therefore, we have been suggested that EVs could contain RNA and that NPCE cells shuttle RNA to TM cells via exosome transfer. Total RNA derived from NPCE EVs was analysed using Agilent Bioanalyzer. After filtering out signals of low intensity, we detected, on average, 696 miRNAs per sample. As expected, EV samples contained little quantities of 18S and 28S ribosomal RNA. To identify potential miRNAs that regulate genes involved in the regulation of the Wnt pathway, the miRNAs found in the microarray analysis were matched to previously published data of miRNAs that were reported in the AH of cataract patients.^33^ When comparing our data *vs.* AH data, we found that over two‐thirds of the most overlapped AH miRNAs detected in five studies were also presented in the NPCE EVs. By generating a Venn diagram, we found 77 miRNAs which were common between NPCE EVs and AH miRNAs (Figure 2A,B). As shown in Figure 2C, the most abundant of these 77 miRNAs identified in the NPCE EV samples was miR‐21, followed by miR‐638, let‐7a, miR‐100 and miR‐16. Many of the abundant miRNAs in EVs isolated from NPCE cells have been reported to regulate Wnt signalling (Table 2), and other miRNAs such as miR‐29, miR‐17 and miR‐21 have been related to the modulation of collagen synthesis. These results suggest that EVs carry miRNAs that have regulatory functions and can perhaps give rise to proteins or perform regulation on the recipient cells.

**Table 2:** Effect of MiRNA detected in NPCE cell line‐derived EVs in the regulation of WNT/β‐catenin signalling pathway

miRNA	Wnt‐signaling target	Inhibits/activates Wnt signaling pathway	References
miR‐21	DKK2, TGFβR2, Wnt1	Activates or Inhibits	51, 54, 55
miR‐638	FZD7	Inhibits	56
miR‐100	DKK1, ZNRF3	Activates	57
miR‐125	DKK3, ZNRF3, RNF43, APC2	Activates	57
miR‐23a	FZD5	Inhibits	58
miR‐29a	sFRP‐2, DKK1	Activates	59
miR‐15b	Axin2	Activates	60
miR‐29b	TCF7L2, BCL9L, SNAI1	Inhibits	61
miR‐146a	ZNRF3	Activates	62
miR‐20a/19b	E2F1, HIPK1	Activates	63
miR‐130a	RUNX3	Activates	64
miR‐27a‐3p	SFRP1	Activates	65
miR‐365b	Wnt5a	Inhibits	66
miR‐25	β‐catenin	Inhibits	67
miR‐181a	WIF1	Activates	68

### 3.4 Characterization of EVs isolated from primary NPCE cells

**Figure 3:**
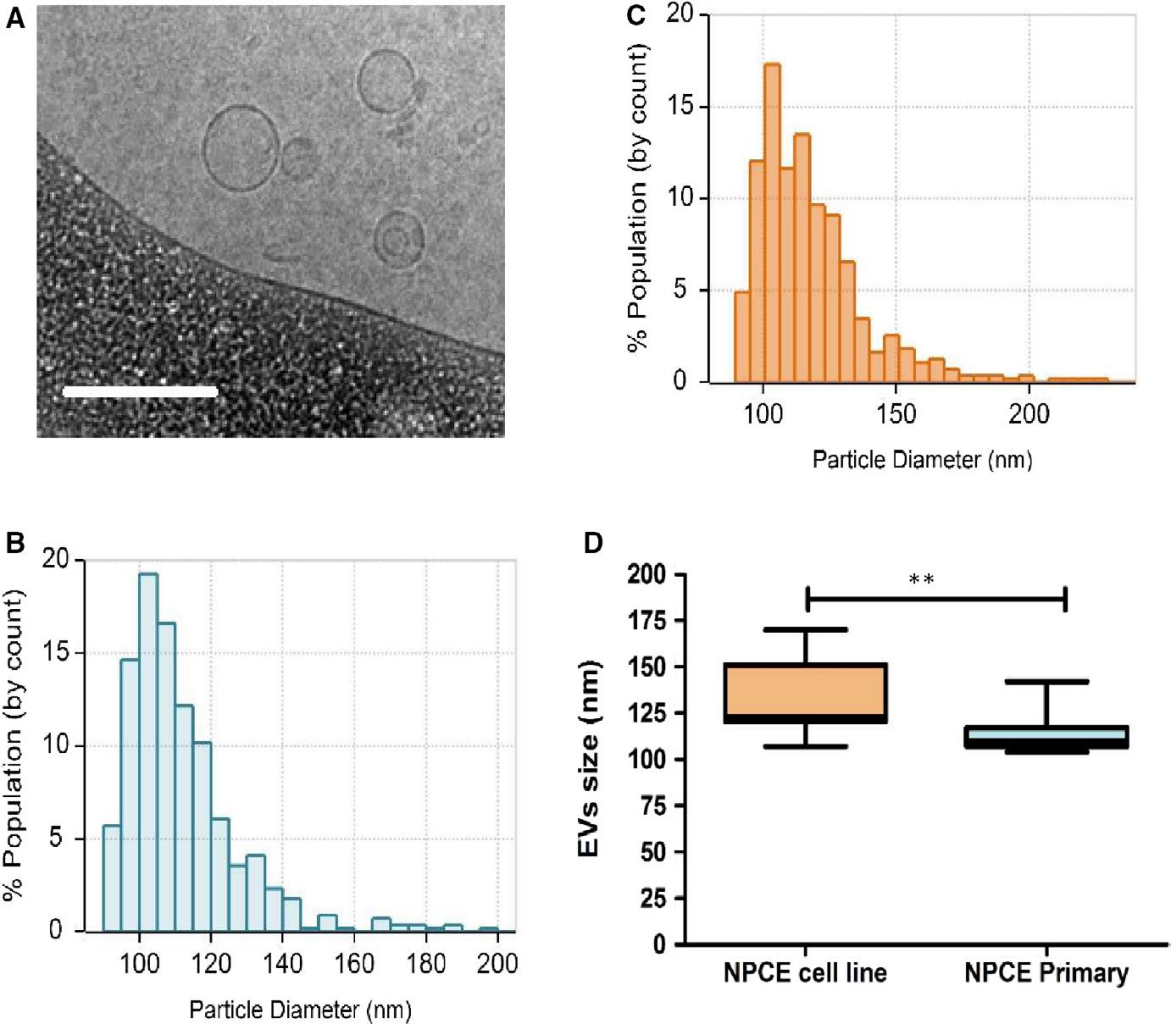
Identification and characterization of EVs. Condition medium from primary NPCE cells and NPCE cell line was collected. EVs were isolated and characterized by cryo‐TEM and TPRS analysis. A, Representative cryo‐TEM image of EVs from primary NPCE cell culture. The scale bar is 200 nm. B, Particle size measurement of the primary NPCE EVs detected by TRPS. C, Particle size measurement of NPCE cell line‐derived EVs detected by TRPS. Histograms are from more than 500 individual EV events. D, Box plot showing the particle size distribution of the EVs derived from NPCE cell line and from primary NPCE cells detected by TRPS. Data are means ± SEM of 10 independent measurements (**P < .01, unpaired Student's test)

To analyse EVs isolated from primary NPCE cells, cryo‐TEM and TRPS were performed. Using the cryo‐TEM approach, we observed that primary NPCE cells generated typical round shape heterogeneous membrane encapsulated vesicles (Figure 3A). The round‐shaped primary EVs were consistent with morphology observations made previously for NPCE cell line‐derived EVs.^17^ The average size of primary NPCE EVs measured by TRPS was comparatively smaller than that obtained for EVs derived from NPCE cell line (113.73 ± 10.64 vs 131.64 ± 18.83, respectively *P* < .01) (Figure 3B,C). EV size range was 30‐150nm, similar to NPCE cell line (36 −174nm) (Figure 3D).

### 3.5 EVs released from primary NPCE cells modulate Wnt signalling pathway in primary TM cells

**Figure 4:**
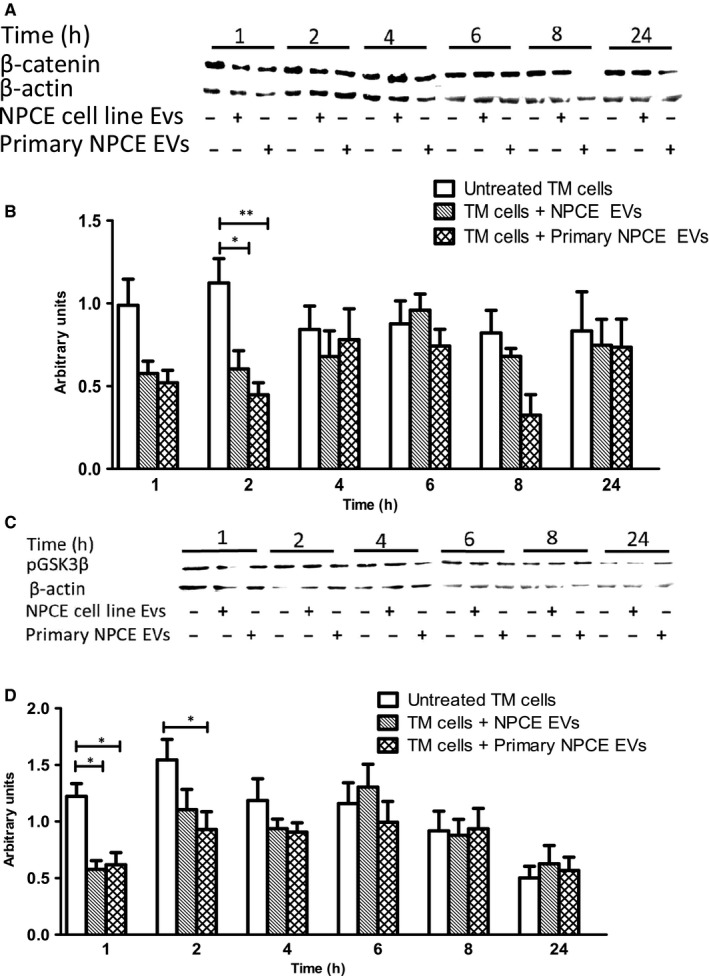
Effect of EVs isolated from primary NPCE cells and NPCE cell line on β‐catenin and GSK3β protein expression in primary TM cells. Primary TM cells were treated with NPCE primary or cell line‐derived EVs and the effect of EVs on protein level of β‐catenin and pGSK3β in TM cells was detected by Western blotting at the indicated time‐points. A, Representative Western blots showing the levels of β‐catenin or (C) pGSK3β protein, phosphorylated at Ser9 in primary TM cells following EV treatment. B, The graph shows the densitometry quantification for the expression of β‐catenin or (D) the expression of pGSK3β in primary TM cells. Data presented as mean ± SEM of three independent experiments. Asterisks indicate statistically significant differences from untreated control (*P < .05, **P < .01 in two‐way ANOVA with Bonferroni's test)

Based on our previous findings that NPCE and TM cell lines establish an EV‐mediated crosstalk leading to an inhibition of Wnt signalling in TM cells, we expected that primary NPCE EVs would also suppress Wnt signalling pathway in primary TM cells. To test whether this trend is observed in primary cells, primary TM cells were co‐cultured with either EVs secreted by primary NPCE cells or EVs secreted by NPCE cell line, and the expression level of specific Wnt signalling proteins was determined by Western blotting. Consistent with the results in the TM cell line, EVs released by both primary NPCE cells and NPCE cell line showed diminished pGSK3β phosphorylation and decreased cytosolic levels of β‐catenin in primary TM cells. We observed a twofold decrease in total β‐catenin protein levels after 2 hours of primary NPCE or cell line exosomal treatment (*P* < .01, *P* < .05) (Figure 4A,B) and significantly lower protein levels of pGSK3β after 1‐2 hours of treatment with either NPCE primary or cell line released EVs (*P* < .01, *P* < .05) (Figure 4C,D). No significant changes in these protein levels were seen after more than a 2‐hour incubation, compared with untreated TM control cells.

### 3.6 Effect of NPCE EVs on PDK1/Akt/mTOR signalling pathway in a TM cell line

**Figure 5:**
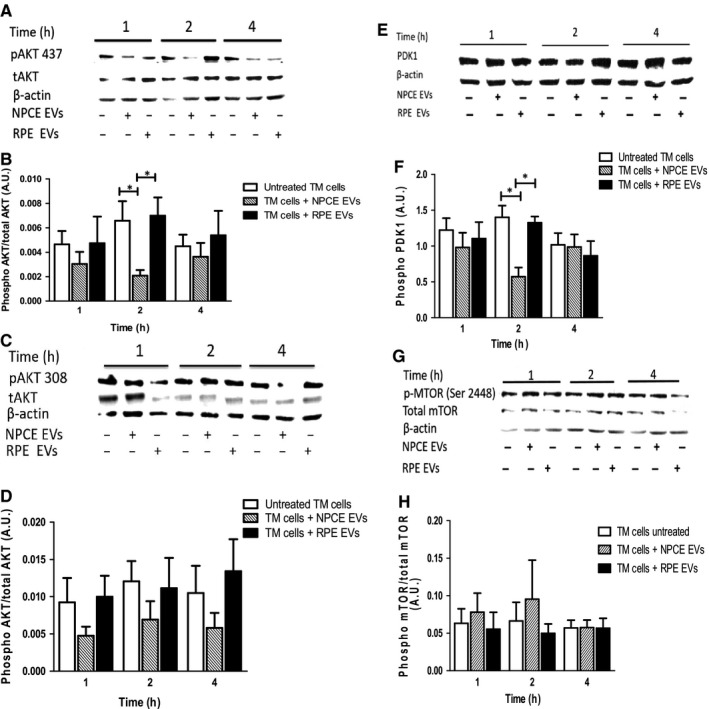
Effect of NPCE cell line EVs on activity of AKT, PDK1 and mTOR proteins. Experimental conditions included untreated TM cells and TM cells treated with either control (RPE EVs) or NPCE EVs for the indicated times. Total cell lysates were prepared from TM cells and subjected to the western blot analyses with p‐AKT, t‐AKT, p‐PDK1, p‐mTORC1 and t‐mTORC1 antibodies. Actin was used to verify equal loading. Representative western blots demonstrate (A) pAKT 473, (c) pAKT 308, (E) p‐PDK1 (Ser 241) and (G) p‐mTORC1 (Ser 2448) protein levels. Densitometric evaluation of (B) p‐AKT 473, (D) p‐AKT 308, (F) p‐PDK1 and (H) t‐mTORC1 protein expression. For all graphs, data are means ± SEM of at least three independent experiments. (*P < .05, **P < .01 and ***P < .001 in two‐way ANOVA with Bonferroni's test)

Inhibitory serine‐phosphorylation is the most frequently examined mechanism that regulates the activity of GSK3. It is well established that AKT inhibits GSK3 kinase activity via phosphorylation of Ser‐9 in GSK3β.^35^ A possible mechanism by which the NPCE EVs can mediate the inhibition of Wnt signalling pathway in TM cells is by decreasing the protein level of phosphorylated Akt protein. Akt has been shown to phosphorylate and inactivate GSK3β, which is required for the deactivation of β‐catenin. Therefore, AKT protein level was measured in protein extracts derived from the TM samples using Western blot analysis with anti‐AKT monoclonal antibody and anti‐β‐actin as control. As the full deactivation of Akt requires de‐phosphorylation at both Thr308 and Ser473,^36^ we examined two‐site phosphorylation in TM cells following NPCE EV treatment at different time‐points (1,2,4 hours). As controls, we used untreated TM cells and TM cells that were exposed to RPE EVs, which we had previously shown to be unable to reduce β‐catenin levels in TM cells. When the TM cells treated with NPCE EVs for 2h, we observed greater than a threefold decrease (*P* < .05) of Akt phosphorylated at Ser473 compared with untreated cells (Figure 5A,B). A trend of lower Akt phosphorylation levels at Thr308 was observed upon NPCE exposure, without reaching significance (Figure 5C,D). No differences were detected in the expression levels of Akt phosphorylation at both the Ser473 and Thr308 positions upon RPE EV exposure, as compared to the untreated control. In the attempt to better define the molecular mechanism underlying the ability of EVs to modulate Akt protein we further investigated the phosphorylation state of proteins that lies upstream (PDK1) and downstream (mTORC1) of Akt in TM cells following EV treatment. In response to NPCE EVs at 2 hours, TM cells exhibited 2.5‐fold (*P* < .05) decreased phosphorylation of PDK1 but pre‐treatment of TM cells with RPE EVs had no effect on the amount of PDK1 at the respective time‐point (Figure 5E,F). Next, we examined how the presence or absence of NPCE‐derived EVs affects mTORC1 phosphorylation. Results revealed that no significant differences were detected following NPCE and RPE EV treatment regarding their effects on phosphorylation of the mTORC1 at Ser2448 (Figure 5G,H). Collectively, these results suggest that NPCE EVs seem likely to play some role in phosphorylation of Akt and Pdk1 proteins. However, as EVs were unable to inhibit mTORC1 phosphorylation by Akt protein, and Akt was partially activated by phosphorylation of T308, Akt protein in our system cannot account for the reduced phosphorylation of p‐GSK3β in TM cells.

### 3.7 Effect of NPCE EVs on PP2A protein levels in a TM cell line

**Figure 6:**
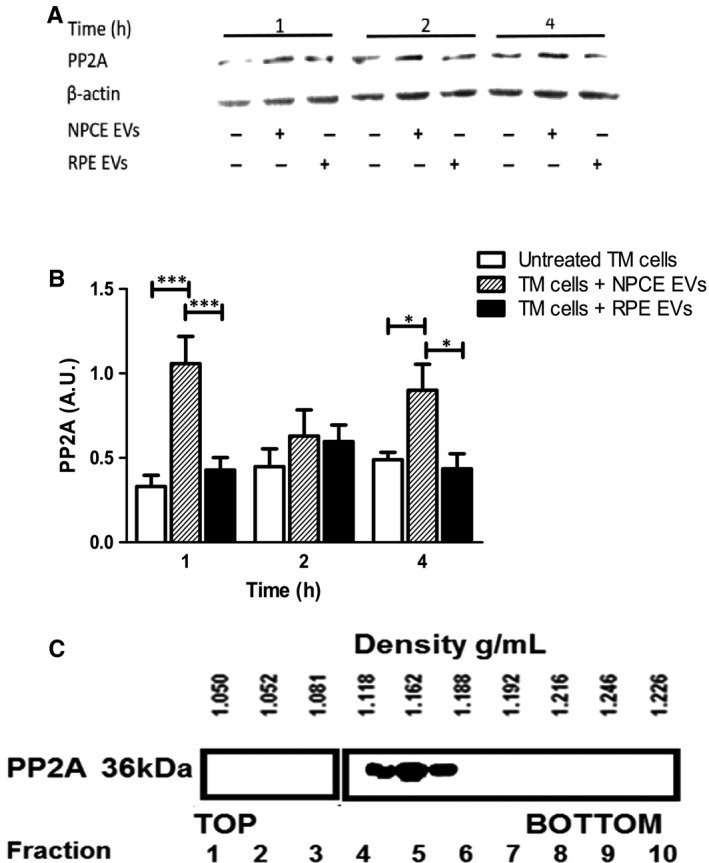
Expression of PP2A in TM cells and NPCE EVs. Western blot showing increased PP2A protein levels in the TM cells after NPCE EV exposure. A, Western blot representative images. B, Quantification of PP2A in TM cells exposed to NPCE or RPE EVs or untreated cells. Results are representative of three independent experiments (*P < .05; ***P < .001 in two‐way ANOVA with Bonferroni's test). C, NPCE EVs were floated into a linear sucrose gradient (0.25‐2.5 mol/L) and then subjected to overnight centrifugation. Gradient fractions were collected and analysed by Western blot analysis using PP2A antibody

PP2A catalyses dephosphorylation of Akt at Ser473 to partially deactivate it and dephosphorylates GSK3β at Ser9 to activate it.^37^, ^38^ Because NPCE EVs induced deactivation of Akt kinase at Ser473 position only, and activated GSK3β by lowering its phosphorylation at Ser9, we next tested whether NPCE EVs regulate PP2A activity in TM cells. To investigate the effect of EVs on PP2A levels in TM cells, the cells were grown in media with either NPCE EVs or RPE EVs. Untreated cells served as control. Proteins lysates were extracted and PP2A expression was detected by Western blot. Results revealed that pretreated TM cells with NPCE EVs resulted in a greater than a twofold (*P* < .001) increase in the PP2A protein levels as compared to control cells (Figure 6A,B). Next, we investigated whether the NPCE EVs are positive for PP2A protein. Western blot analysis of density gradient fractions confirmed the presence of PP2A in NPCE EVs (Figure 6C). We observed, using a continuous sucrose gradient, that most of the PP2A was recovered in fractions 4 to 6, ranging from densities of 1.18‐1.188 g/mL, corresponding to the density range classically assigned to EVs.^39^ These results suggest that NPCE cells secrete EVs containing PP2A protein and thus may be responsible for the elevation levels of PP2A protein in TM cells and downregulation of Wnt signalling by PP2A protein delivery and activation of GSK3β in recipient cells.

### 3.8 NPCE EV effect on collagens protein levels in TM cells

**Figure 7:**
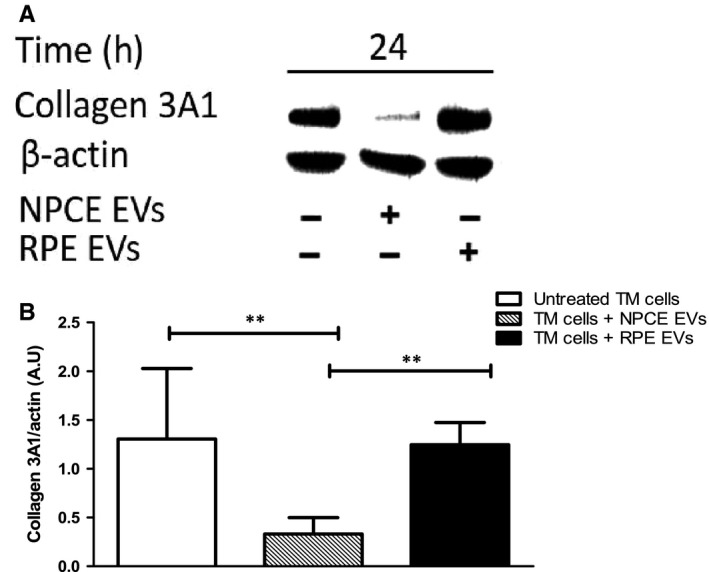
Expression of type III collagen in TM cells following exposure to NPCE cell line‐derived EVs. A, Representative Western blot showing collagen 3A1 expression in TM cells exposed to NPCE or RPE EVs or untreated cells. B, Quantitative analysis of collagen levels relative to β‐actin in response to NPCE and RPE EVs. Results are representative of three independent experiments. (**P < .01 in one‐way ANOVA with post‐Tukey's test)

Trabecular meshwork tissue consists of various extracellular matrix (ECM) proteins, including collagen (types I, III, IV and V), proteoglycans and laminin.^40^ Changes in ECM are thought to have a role in the increased out‐flow resistance of the TM in POAG.^41^ Activation of the Wnt signalling pathway in humans has been shown to promote fibrosis of various organs.^42^ We have been suggested that down‐regulation of Wnt/β‐catenin signalling pathway by NPCE EVs would reduce collagen protein expression in TM cells. The effect of NPCE‐derived EVs on the levels of collagen IIIA (Col3A) in TM cells was investigated by Western blotting analysis with anti‐Col3a monoclonal Ab. As shown in Figure 7A,B, treatment with NPCE EVs clearly decreased the collagen III expression in TM cells after 24 hours incubation of these cells with NPCE EVs. When normalized to the loading control, expression of collagens in TM cells were approximately threefold (*P* < .01) lower than untreated control or RPE EV‐treated cells.

## 4 DISCUSSION

In the present study, we explore the idea that NPCE‐derived EVs act as intercellular communicators to facilitate direct transfer of biological active cargo carried by NPCE EVs to TM cells. We aimed to gain a better understanding on how the NPCE EVs influence the TM Wnt signalling pathway. We focused on investigating the effect of NPCE EVs on expression of Akt and PP2A proteins, which control the Wnt pathway, by regulation of GSK3β phosphorylation state. Currently, there is no information about the production of EVs by NPCE cells, and no evaluation of NPCE‐derived exosome content has been performed. We characterized protein and miRNA content of EVs released by NPCE cells to shed more light on their molecular components that may be involved in Wnt regulation.

Our present findings demonstrate that EVs derived from NPCE cell line can modulate the Wnt signalling in TM cell line, having the same trend and power as primary NPCE cells’ EVs. When we treated primary TM cells with NPCE EVs (from primary or cell line) a similar Wnt signalling attenuation was found. TM cells treated with the NPCE EVs displayed decreased Akt473 phosphorylation with no apparent change at Thr308. Full activation requires both sites to be phosphorylated, whereas singly phosphorylated Akt is partially active, suggesting that NPCE EVs may regulate GSK3β serine 9 phosphorylation site by PP2A phosphatase. Consistent with this possibility, we have found that NPCE EVs increase PP2A expression in TM cells. Furthermore, we showed that NPCE EVs carry PP2A phosphatase. Proteomic exploration of the NPCE EVs revealed that many previously identified AH proteins were found to be enriched in NPCE EV samples, including proteins related to adhesion (CD44, CD59), antigen presentation (β‐2‐microglobulin), cytoskeleton (actin, actinin, tropomyosin α), oxidative stress response (glutathione S‐transferase, peroxiredoxin‐1, SOD), protein synthesis regulation, signal transduction proteins and enzymes. The resulting miRNAs’ profiles of NPCE‐derived EVs demonstrated the presence of numerous miRNAs, which were previously shown to be involved in regulation of TM cellular processes. For example, in TM cells, introduction of mir‐29, resulted in down‐regulation of multiple ECM components, including collagens and genes involved in ECM deposition.^43^ We found that members of 29‐miRNA family were abundant in NPCE exosomal samples and that treatment of TM cells with NPCE EVs significantly decreased the expression of the COL3A1. In addition, fifteen miRNAs were found to be either potent activators or inhibitors of Wnt signalling.

Taken together, these data indicate that EVs released by NPCE cells may regulate Wnt signalling in TM tissue. Regarding the Wnt signalling pathway attenuation in TM cells by NPCE‐derived EVs, the cell lines used are relevant as they match primary cells. The exact mechanism that underlies the inhibition of Wnt signalling following NPCE exosome treatment in TM cells remains incompletely understood. We propose that one potential mechanism for the observed inhibition of GSK3β is through transfer of PP2A phosphatase to TM cells. Phospho‐GSK3β is a substrate for PP2A, and dephosphorylation of GSK3β by PP2A may lead to a reduction in β‐catenin accumulation and a decrease in Wnt signalling pathway activity. Another possible mechanism involved in mediating exosome Wnt inhibition effect could be via the exosome delivery of miRNAs. The presence of 89% identified AH miRNAs in NPCE EVs suggests that NPCE tissue may be the pivotal source of miRNAs found in AH. Our understanding is that exosome miRNAs may target genes in TM cells and contribute to the ocular drainage system homeostasis by modulating levels of β‐catenin and levels of several ECM proteins such as collagen. We believe that exosomal cargo may vary in response to different physiological or pathological conditions, and AH out‐flow system may utilize multiple miRNAs, creating a sophisticated balance between activation and repression of Wnt signalling.

Accumulating evidence suggests that the Wnt pathway is a major signalling pathway contributing to the TM AH drainage resistance.^44^, ^45^ It was shown that Wnt antagonist increases out‐flow facility in perfused human anterior segments.^20^ Wnt signalling components were found in different ocular drainage tissues, among them sFRP‐1, an antagonist of the Wnt signalling pathway. Inhibition of the Wnt pathway using sFRP1 increases IOP in vivo in mice animal models.^46^ Wnt signalling pathway has a complex relation with the ECM. It intersects with TGF‐beta pathway to regulate ECM gene expression in multiple cell types, and plays a role in ECM assembly.^47^ Evidence from open‐angle glaucoma studies suggests a link between Wnt inhibition and increased TM cell stiffness.^48^ It is therefore possible that dysregulation of Wnt signalling pathway may cause ECM abnormalities in the TM. Our results showed reduction in the main fibrous ECM protein and proteins related to the Wnt pathway following treatment with NPCE EVs isolated under normal condition. However, the role of EVs produced under stress condition that might mimic elevated ocular pressure needs to be further investigated.

Recent studies have suggested that miRNAs participate in regulating Wnt pathway by targeting multiple Wnt signalling pathway components.^49^ Among the top 27 most abundant miRNAs detected in NPCE exosome samples and AH, most have important function in Wnt signalling pathway regulation. For example, NPCE‐derived EVs contain significant amounts of miR‐638, which was linked to the down‐regulation of the Wnt/β‐catenin signalling pathway.^14^ In addition, miR‐21 was detected in NPCE cells derived EVs at high concentration compared with other miRNAs detected. miR‐21 is indeed involved in the Wnt/β‐catenin signalling pathway through its upstream target genes and positive regulation of Wnt, thereby activating the Wnt/β‐catenin signalling pathway and causing β‐catenin expression to increase.^50^ miR‐21 could negatively modulate the activity of Wnt/β‐catenin signalling via targeting Wnt‐1, which likely accounts for the Wnt/β‐catenin cascade activation.^51^ Furthermore, miR‐21 was shown to target TIMP3, a tissue inhibitor of MMPs, promoting MMP activity, leading to degradation of ECM.^52^ Another recent study on human tissues suggests that miR‐21 promotes ECM degradation through inhibiting autophagy via the PTEN/Akt/mTOR signalling pathway.^53^

Along the cascade of PI3K/AKT signalling pathway, potential elements involved in the regulation of WNT pathway is PP2A. PP2A was found to be significantly higher in AH samples from the POAG group as compared to a cataract group.^28^ After treatment with DEX, TGFβ2 alkaline phosphatases activity was reported to be increased in TM cells. Alkaline phosphatases activity was also found to be significantly higher in the intact TM tissues from glaucomatous patients.^29^ PP2A has been shown to negatively regulate signalling networks such as the Wnt pathway. Yokoyama et al revealed that knockdown of the PP2Ac catalytic subunit led to increases in phosphorylated‐GSK3β.^27^ Mitra et al confirmed this finding and show that PP2A‐mediated dephosphorylation of GSK3β occurs through recruitment of two heat shock proteins. These results support a potential role of PP2A in dephosphorylating, and thereby activating, GSK3β with resultant phosphorylation of β‐catenin leading to its destruction.^26^

In conclusion, our findings propose exosome‐mediated crosstalk between NPCE cells and TM cells that affect the behaviour of target TM cells through modulation of Wnt signalling pathway and ECM remodelling, probably, by the transfer of proteins and RNA cargo. Exploration of the precise physiological roles of NPCE EVs may allow the development of novel therapeutic approaches, with minimally invasive procedures.

## CONFLICT OF INTEREST

The authors declare that they have no conflicts of interest.

## Data Availability

The data that support the findings of this study are available from the corresponding author upon reasonable request.

## References

[1] Bourne RRA , Taylor HR , Flaxman SR , et al. Number of people blind or visually impaired by glaucoma worldwide and in world regions 1990–2010: a meta‐analysis. PloS One. 2016;11(10):e0162229. 27764086 10.1371/journal.pone.0162229 PMC5072735

[2] Vajaranant TS , Wu S , Torres M , Varma R . The changing face of primary open‐angle glaucoma in the United States: demographic and geographic changes from 2011 to 2050. Am J Ophthalmol. 2012;154(2):303‐314.e3. 22541661 10.1016/j.ajo.2012.02.024 PMC3401269

[3] Weinreb RN , Aung T , Medeiros FA . The pathophysiology and treatment of glaucoma: a review. JAMA. 2014;311(18):1901‐1911. 24825645 10.1001/jama.2014.3192 PMC4523637

[4] McMonnies CW . Glaucoma history and risk factors. J Optom. 2017;10(2):71‐78. 27025415 10.1016/j.optom.2016.02.003 PMC5383456

[5] Goel M , Picciani RG , Lee RK , Bhattacharya SK . Aqueous humor dynamics: a review. Open Ophthalmol J. 2010;4:52‐59. 21293732 10.2174/1874364101004010052 PMC3032230

[6] O’Callaghan J , Cassidy PS , Humphries P . Open‐angle glaucoma: therapeutically targeting the extracellular matrix of the conventional outflow pathway. Expert Opin Ther Targets. 2017;21(11):1037‐1050. 28952395 10.1080/14728222.2017.1386174

[7] Abu‐Hassan DW , Acott TS , Kelley MJ . The trabecular meshwork: a basic review of form and function. J Ocular Biol. 2014;2(1). 10.13188/2334-2838.1000017 PMC4209746 25356439

[8] Torrejon KY , Pu D , Bergkvist M , Danias J , Sharfstein ST , Xie Y . Recreating a human trabecular meshwork outflow system on microfabricated porous structures. Biotechnol Bioeng. 2013;110(12):3205‐3218. 23775275 10.1002/bit.24977

[9] Coca‐Prados M , Escribano J . New perspectives in aqueous humor secretion and in glaucoma: the ciliary body as a multifunctional neuroendocrine gland. Prog Retin Eye Res. 2007;26(3):239‐262. 17321191 10.1016/j.preteyeres.2007.01.002

[10] Funato Y , Michiue T , Asashima M , Miki H . The thioredoxin‐related redox‐regulating protein nucleoredoxin inhibits Wnt–β‐catenin signalling through dishevelled. Nat Cell Biol. 2006;8(5):501‐508. 16604061 10.1038/ncb1405

[11] Pattabiraman PP , Epstein DL , Rao PV . Regulation of adherens junctions in trabecular meshwork cells by Rac GTPase and their influence on intraocular pressure. J Ocular Biol. 2013;1(1). 10.13188/2334-2838.1000002 PMC4057051 24932460

[12] Shahidullah M , Al‐Malki WH , Delamere NA . Mechanism of aqueous humor secretion, its regulation and relevance to glaucoma In: Rumelt S, ed. Glaucoma‐Basic and Clinical Concepts. London, UK: IntechOpen; 2011:3‐32.

[13] Dismuke WM , Challa P , Navarro I , Stamer WD , Liu Y . Human aqueous humor exosomes. Exp Eye Res. 2015;132:73‐77. 25619138 10.1016/j.exer.2015.01.019 PMC4352394

[14] Wang L , Yu Z , Wan S , et al. Exosomes derived from dendritic cells treated with Schistosoma japonicum soluble egg antigen attenuate DSS‐induced colitis. Front Pharmacol. 2017;8:651. 28959207 10.3389/fphar.2017.00651 PMC5603738

[15] Yáñez‐Mó M , Siljander PR , Andreu Z , et al. Biological properties of extracellular vesicles and their physiological functions. J Extracell Vesicles. 2015;4(1):27066. 25979354 10.3402/jev.v4.27066 PMC4433489

[16] Pant S , Hilton H , Burczynski ME . The multifaceted exosome: biogenesis, role in normal and aberrant cellular function, and frontiers for pharmacological and biomarker opportunities. Biochem Pharmacol. 2012;83(11):1484‐1494. 22230477 10.1016/j.bcp.2011.12.037 PMC7110994

[17] Gould SJ , Raposo GA . As we wait: coping with an imperfect nomenclature for extracellular vesicles. J Extracell Vesicles. 2013;2(1):20389. 10.3402/jev.v2i0.20389 PMC3760635 24009890

[18] Witwer KW , Théry C . Extracellular vesicles or exosomes? On primacy, precision, and popularity influencing a choice of nomenclature. J Extracell Vesicles. 2019;8(1):1648167. 31489144 10.1080/20013078.2019.1648167 PMC6711079

[19] Lerner N , Avissar S , Beit‐Yannai E . Extracellular vesicles mediate signaling between the aqueous humor producing and draining cells in the ocular system. PloS One. 2017;12(2):e0171153. 28241021 10.1371/journal.pone.0171153 PMC5328276

[20] Wang W‐H , McNatt LG , Pang I‐H , et al. Increased expression of the WNT antagonist sFRP‐1 in glaucoma elevates intraocular pressure. J Clin Invest. 2008;118(3):1056‐1064. 18274669 10.1172/JCI33871 PMC2242621

[21] Ahadome SD , Zhang C , Tannous E , Shen J , Zheng JJ . Small‐molecule inhibition of Wnt signaling abrogates dexamethasone‐induced phenotype of primary human trabecular meshwork cells. Exp Cell Res. 2017;357(1):116‐123. 28526237 10.1016/j.yexcr.2017.05.009 PMC5535738

[22] Caliceti C , Nigro P , Rizzo P , Ferrari R . and Wnt signaling pathways: crosstalk between three major regulators of cardiovascular biology. BioMed Res Int. 2014;2014:1‐8. 10.1155/2014/318714 PMC3932294 24689035

[23] Shen X , Ying H , Yue BY . Wnt activation by wild type and mutant myocilin in cultured human trabecular meshwork cells. PLoS One. 2012;7(9):e44902. 23028669 10.1371/journal.pone.0044902 PMC3441605

[24] Anderson EC , Wong MH . Caught in the Akt: regulation of Wnt signaling in the intestine. Gastroenterology. 2010;139(3):718‐722. 20659460 10.1053/j.gastro.2010.07.012 PMC3037729

[25] Gauthier AC , Liu J . Epigenetics and signaling pathways in glaucoma. BioMed Res Int. 2017;2017:1‐12. 10.1155/2017/5712341 PMC5292191 28210622

[26] Thompson J , Williams C . Protein phosphatase 2A in the regulation of Wnt signaling, stem cells, and cancer. Genes. 2018;9(3):121. 10.3390/genes9030121 PMC5867842 29495399

[27] Yokoyama N , Malbon CC . Phosphoprotein phosphatase‐2A docks to Dishevelled and counterregulates Wnt3a/β‐catenin signaling. J Mol Signal. 2007;2(1):12. 17961225 10.1186/1750-2187-2-12 PMC2211464

[28] Latarya G , Mansour A , Epstein I , et al. Human aqueous humor phosphatase activity in cataract and glaucoma. Invest Ophthalmol Vis Sci. 2012;53(3):1679‐1684. 22323459 10.1167/iovs.11-9120

[29] Xue W , Comes N , Borrás T . Presence of an established calcification marker in trabecular meshwork tissue of glaucoma donors. Invest Ophthalmol Vis Sci. 2007;48(7):3184‐3194. 17591888 10.1167/iovs.06-1403 PMC1994153

[30] Dismuke WM , Klingeborn M , Stamer WD . Mechanism of fibronectin binding to human trabecular meshwork exosomes and its modulation by dexamethasone. PloS One. 2016;11(10):e0165326. 27783649 10.1371/journal.pone.0165326 PMC5081181

[31] Rider MA , Hurwitz SN , Meckes DG Jr . ExtraPEG: a polyethylene glycol‐based method for enrichment of extracellular vesicles. Sci Rep. 2016;6:23978. 27068479 10.1038/srep23978 PMC4828635

[32] Gong F , Wang G , Ye J , Li T , Bai H , Wang W . 14‐3‐3β regulates the proliferation of glioma cells through the GSK3β/β‐catenin signaling pathway. Oncol Rep. 2013;30(6):2976‐2982. 24065186 10.3892/or.2013.2740

[33] Weiske J , Huber O . The histidine triad protein Hint1 interacts with Pontin and Reptin and inhibits TCF–β‐catenin‐mediated transcription. J Cell Sci. 2005;118(14):3117‐3129. 16014379 10.1242/jcs.02437

[34] Chairoungdua A , Smith DL , Pochard P , Hull M , Caplan MJ . Exosome release of β‐catenin: a novel mechanism that antagonizes Wnt signaling. J Cell Biol. 2010;190(6):1079‐1091. 20837771 10.1083/jcb.201002049 PMC3101591

[35] Wu D , Pan W . GSK3: a multifaceted kinase in Wnt signaling. Trends Biochem Sci. 2010;35(3):161‐168. 19884009 10.1016/j.tibs.2009.10.002 PMC2834833

[36] Wu Y , Zu K , Warren MA , Wallace PK , Ip C . Delineating the mechanism by which selenium deactivates Akt in prostate cancer cells. Mol Cancer Ther. 2006;5(2):246‐252. 16505097 10.1158/1535-7163.MCT-05-0376

[37] Beurel E , Grieco SF , Jope RS . Glycogen synthase kinase‐3 (GSK3): regulation, actions, and diseases. Pharmacol Ther. 2015;148:114‐131. 25435019 10.1016/j.pharmthera.2014.11.016 PMC4340754

[38] McCubrey JA , Steelman LS , Bertrand FE , et al. Multifaceted roles of GSK‐3 and Wnt/β‐catenin in hematopoiesis and leukemogenesis: opportunities for therapeutic intervention. Leukemia. 2014;28(1):15‐33. 23778311 10.1038/leu.2013.184 PMC3887408

[39] Théry C , Amigorena S , Raposo G , Clayton A . Isolation and characterization of exosomes from cell culture supernatants and biological fluids. Curr Protoc Cell Biol. 2006;30(1):3.22. 1‐3.22. 29. 10.1002/0471143030.cb0322s30 18228490

[40] Murphy CG , Yun AJ , Newsome DA , Alvarado JA . Localization of extracellular proteins of the human trabecular meshwork by indirect immunofluorescence. Am J Ophthalmol. 1987;104(1):33‐43. 3300351 10.1016/0002-9394(87)90290-x

[41] Acott TS , Kelley MJ . Extracellular matrix in the trabecular meshwork. Exp Eye Res. 2008;86(4):543‐561. 18313051 10.1016/j.exer.2008.01.013 PMC2376254

[42] Enzo M , Rastrelli M , Rossi C , Hladnik U , Segat D . The Wnt/β‐catenin pathway in human fibrotic‐like diseases and its eligibility as a therapeutic target. Mol Cell Ther. 2015;3(1):1. 26056602 10.1186/s40591-015-0038-2 PMC4452070

[43] Luna C , Li G , Qiu J , Epstein DL , Gonzalez P . Role of miR‐29b on the regulation of the extracellular matrix in human trabecular meshwork cells under chronic oxidative stress. Mol Vis. 2009;15(263–67):2488‐2497. 19956414 PMC2786891

[44] Villarreal G , Chatterjee A , Oh SS , Oh D‐J , Kang MH , Rhee DJ . Canonical Wnt signaling regulates extracellular matrix expression in the trabecular meshwork. Invest Ophthalmol Vis Sci. 2014;55(11):7433‐7440. 25352117 10.1167/iovs.13-12652 PMC4238318

[45] Webber HC , Bermudez JY , Sethi A , Clark AF , Mao W . Crosstalk between TGFβ and Wnt signaling pathways in the human trabecular meshwork. Exp Eye Res. 2016;148:97‐102. 27091054 10.1016/j.exer.2016.04.007 PMC5310225

[46] Kaufman PL , Rasmussen CA . Advances in glaucoma treatment and management: outflow drugs. Invest Ophthalmol Vis Sci. 2012;53(5):2495‐2500. 22562850 10.1167/iovs.12-9483m PMC4898880

[47] Tan J , Tong BD , Wu YJ , Xiong W . MicroRNA‐29 mediates TGFbeta1‐induced extracellular matrix synthesis by targeting wnt/beta‐catenin pathway in human orbital fibroblasts. Int J Clin Exp Pathol. 2014;7(11):7571‐7577. 25550793 PMC4270536

[48] Morgan JT , Raghunathan VK , Chang Y‐R , Murphy CJ , Russell P . The intrinsic stiffness of human trabecular meshwork cells increases with senescence. Oncotarget. 2015;6(17):15362. 25915531 10.18632/oncotarget.3798 PMC4558157

[49] Peng Y , Zhang X , Feng X , Fan X , Jin Z . The crosstalk between microRNAs and the Wnt/β‐catenin signaling pathway in cancer. Oncotarget. 2017;8(8):14089‐14106. 27793042 10.18632/oncotarget.12923 PMC5355165

[50] Wu D , Shi M , Fan XD . Mechanism of miR‐21 via Wnt/β‐catenin signaling pathway in human A549 lung cancer cells and Lewis lung carcinoma in mice. Asian Pac J Trop Med. 2015;8(6):479‐484. 26194834 10.1016/j.apjtm.2015.05.003

[51] Hashimi ST , Fulcher JA , Chang MH , Gov L , Wang S , Lee B . MicroRNA profiling identifies miR‐34a and miR‐21 and their target genes JAG1 and WNT1 in the coordinate regulation of dendritic cell differentiation. Blood. 2009;114(2):404‐414. 19398721 10.1182/blood-2008-09-179150 PMC2927176

[52] Gabriely G , Wurdinger T , Kesari S , et al. MicroRNA 21 promotes glioma invasion by targeting matrix metalloproteinase regulators. Mol Cell Biol. 2008;28(17):5369‐5380. 18591254 10.1128/MCB.00479-08 PMC2519720

[53] Wang W‐J , Yang W , Ouyang Z‐H , et al. MiR‐21 promotes ECM degradation through inhibiting autophagy via the PTEN/akt/mTOR signaling pathway in human degenerated NP cells. Biomed Pharmacother. 2018;99:725‐734. 29710470 10.1016/j.biopha.2018.01.154

[54] Kawakita A , Yanamoto S , Yamada S , et al. MicroRNA‐21 promotes oral cancer invasion via the wnt/β‐catenin pathway by targeting DKK2. Pathol Oncol Res. 2014;20(2):253‐261. 23999978 10.1007/s12253-013-9689-y

[55] Yu Y , Kanwar SS , Patel BB , et al. MicroRNA‐21 induces stemness by downregulating transforming growth factor beta‐receptor 2 (TGFβR2) in colon cancer cells. Carcinogenesis. 2011;33(1):68‐76. 22072622 10.1093/carcin/bgr246 PMC3276336

[56] Chen Z , Duan X . hsa_circ_0000177‐miR‐638‐FZD7‐wnt signaling cascade contributes to the malignant behaviors in glioma. DNA Cell Biol. 2018;37(9):791‐797. 30010402 10.1089/dna.2018.4294

[57] Lu Y , Zhao X , Liu Q , et al. lncRNA MIR100HG‐derived miR‐100 and miR‐125b mediate cetuximab resistance via wnt/ß‐catenin signaling. Nat Med. 2017;23(11):1331‐1341. 29035371 10.1038/nm.4424 PMC5961502

[58] Listing H , Mardin WA , Wohlfromm S , Mees ST , Haier J . MiR‐23a/‐24‐induced gene silencing results in mesothelial cell integration of pancreatic cancer. Br J Cancer. 2015;112(1):131‐139. 25422915 10.1038/bjc.2014.587 PMC4453619

[59] Kapinas K , Kessler C , Ricks T , Gronowicz G , Delany AM . miR‐29 modulates wnt signaling in human osteoblasts through a positive feedback loop. J Biol Chem. 2010;285(33):25221‐25231. 20551325 10.1074/jbc.M110.116137 PMC2919085

[60] Guo X , Zhang Y , Li J , Liao M . miR‐15b facilitates the progression of colorectal cancer via targeting β‐catenin and Axin2. Int J Clin Exp Pathol. 2016;9(9):8990‐8996.

[61] Subramanian M , Rao SR , Thacker P , Chatterjee S , Karunagaran D . MiR‐29b downregulates canonical wnt signaling by suppressing coactivators of ß‐catenin in human colorectal cancer cells. J Cell Biochem. 2014;115(11):1974‐1984. 24913975 10.1002/jcb.24869

[62] Zhou C , Jiang C , Zong Z , Lin J , Lao L . miR‐146a promotes growth of osteosarcoma cells by targeting ZNRF3/GSK‐3ß/ß‐catenin signaling pathway. Oncotarget. 2017;8(43):74276‐74286. 29088784 10.18632/oncotarget.19395 PMC5650339

[63] Wu Q , Yang Z , Wang F , et al. MiR‐19b/20a/92a regulates the self‐renewal and proliferation of gastric cancer stem cells. J Cell Sci. 2013;126:4220‐4229. 23868977 10.1242/jcs.127944

[64] Xu N , Shen C , Luo Y , et al. Upregulated miR‐130a increases drug resistance by regulating RUNX3 and wnt signaling in cisplatin‐treated HCC cell. Biochem Biophys Res Commun. 2012;425(2):468‐472. 22846564 10.1016/j.bbrc.2012.07.127

[65] Qiao B , He B , Cai J , Tao Q , King‐Yin LA . MicroRNA‐27a‐3p modulates the wnt/β‐catenin signaling pathway to promote epithelial‐mesenchymal transition in oral squamous carcinoma stem cells by targeting SFRP1. Sci Rep. 2017;7:44688‐44688. 28425477 10.1038/srep44688 PMC5397903

[66] Wang Y , Xu C , Wang Y , Zhang X . MicroRNA‐365 inhibits ovarian cancer progression by targeting Wnt5a. Am J Cancer Res. 2017;7(5):1096‐1106. 28560060 PMC5446477

[67] Anton R , Chatterjee SS , Simundza J , Cowin P , DasGupta R . A systematic screen for micro‐RNAs regulating the canonical wnt pathway. PLoS ONE. 2011;6(10):1‐12. 10.1371/journal.pone.0026257 PMC3197157 22043311

[68] Lyu X , Li J , Yun X , et al. miR‐181a‐5p, an inducer of wnt‐signaling, facilitates cell proliferation in acute lymphoblastic leukemia. Oncol Rep. 2017;37(3):1469‐1476. 28184923 10.3892/or.2017.5425

